# Plasma levels of EPA and DHA after ingestion of a single dose of EPA and DHA ethyl esters

**DOI:** 10.1002/lipd.12417

**Published:** 2024-09-19

**Authors:** Henrieke Marie‐Luise Schmieta, Theresa Greupner, Inga Schneider, Sonja Wrobel, Vanessa Christa, Laura Kutzner, Andreas Hahn, William S. Harris, Nils Helge Schebb, Jan Philipp Schuchardt

**Affiliations:** ^1^ Institute of Food and One Health Leibniz University Hannover Hannover Germany; ^2^ Food Chemistry, Faculty of Mathematics and Natural Sciences University of Wuppertal Wuppertal Germany; ^3^ The Fatty Acid Research Institute Sioux Falls South Dakota USA; ^4^ Department of Internal Medicine, Sanford School of Medicine University of South Dakota Sioux Falls South Dakota USA

**Keywords:** bioavailability, docosahexaenoic acid, eicosapentaenoic acid, kinetics, oral single dose, polyunsaturated fatty acid metabolism

## Abstract

Omega‐3 polyunsaturated fatty acids (n3 PUFA), specifically eicosapentaenoic acid (EPA, 20:5n3), and docosahexaenoic acid (DHA, 22:6n3), are essential for maintaining health. To better understand their biology, it is important to define their bioavailability. The aim of this cross‐over study was to investigate and compare the acute effects on plasma EPA and DHA levels after single doses of EPA oil (99% pure) and DHA (97% pure) ethyl esters. Twelve men aged 20–40 years with a body‐mass‐index of 20–27 kg/m^2^ and low fish consumption were recruited. Several measures (e.g., 4‐week run‐in period, standardized diet, and blood collection protocols) were taken to reduce the inter‐individual variability of plasma fatty acids levels. Using a cross‐over design, the subjects received 2.2 g of EPA in the first test period and 2.3 g of DHA in the second. The test periods were separated by 2 weeks. Blood samples were taken before dosing and after 2, 4, 6, 8, 12, 24, 48, and 72 h. The mean ± SE maximum concentrations for EPA were higher than for DHA (115 ± 11 μg/mL vs. 86 ± 12 μg/mL; *p* = 0.05). The mean ± SE incremented area under the plasma concentration curve over 72 h for EPA (2461 ± 279 μg/mL) was 2.4 times higher (*p* < 0.001) than that for DHA (1021 ± 170 μg/mL). The mean ± SE half‐life was for EPA and DHA was 45 ± 8 and 66 ± 12 h. Our results indicate that EPA administration in single doses leads to higher circulating plasma levels of EPA compared to an effect of an equivalent dose of DHA on DHA plasma levels.

**Abbreviations**
aLNA: alpha‐linolenic acid
ARA: arachidonic acid
iAUC: incremental area under the curve
BMI: body mass index
CVD: cardiovascular disease
DHA: docosahexaenoic acid
DPAn3: docosapentaenoic acid n3
DPAn6: docosapentaenoic acid n6
EE: ethyl esters
EMA: european medicines agency
EPA: eicosapentaenoic acid
FA: fatty acid
FAME: fatty acid methyl esters
FID: flame ionization detector
FO: fish oil
GC: gas chromatography
LA: linoleic acid
LDL: low‐density lipoprotein
LLOQ: lower limit of quantification
LC: long chain
MUFA: monounsaturated fatty acids
n3: omega‐3
n6: omega‐6
n.s.: not significant
PUFA: polyunsaturated fatty acids
rTAG: re‐esterified triacylglycerol
SBP: systolic blood pressure
SD: standard deviation
SDA: stearidonic acid
SE: standard error
SFA: saturated fatty acids
TAG: triacylglycerol

## INTRODUCTION

Omega‐3 (n3) polyunsaturated fatty acids (PUFA)—especially eicosapentaenoic acid (EPA, 20:5n3) and docosahexaenoic acid (DHA, 22:6n3)—are important for maintaining health (Djuricic & Calder, 2021). Higher blood levels of n3 PUFAs are associated with lower risk for cardiovascular disease and mortality (Harris et al., 2017; Harris et al., 2021), reduced inflammatory status (Calder, 2014, 2015), as well as slower angiogenesis, tumor growth, and metastasis (Zhang et al., 2013).

In natural fish oils (FO), EPA and DHA are mainly present in triacylglycerols (TAGs) and to a lesser extent as free FA. In refined, concentrated FO, EPA and DHA are re‐esterified to re‐constituted TAGs (rTAGs), whereas most highly concentrated products with EPA or DHA concentrations ≥90%, contain EPA and DHA as ethyl esters (EE). Besides health effects, it is important to understand the bioavailability and kinetics of different n3 PUFA formulations, which is dependent on the chemical form, dosage and formulation (i.e., galenics), food matrix effects (fat content, other food components) and metabolism, including degradation (beta‐oxidation), transfer from plasma to tissues, and transformation (conversion, retro‐conversion) pathways.

The term “kinetic” describes the rate and extent to which a substance is absorbed by the intestinal tract and enters blood circulation after ingestion of a defined dose. It is important to note that high‐sensitive EPA and DHA kinetics requires the use of labeled compounds. With “natural” unlabeled FA, it is not possible to differentiate between EPA and DHA ingested from those already existing in the body. Moreover, EPA and DHA partly undergo rapid metabolism and are quickly integrated into cell membranes. Considering the aforementioned constraints, several crossover studies have examined the kinetic or relative bioavailability of non‐isotopic n3 PUFA following a single‐dose intake (Kang et al., 2023; Schneider et al., 2011; Schuchardt et al., 2011). Here, we use the term “plasma kinetic” to describe the post‐absorptive levels of EPA and DHA in plasma.

The aim of this cross‐over study was to investigate and compare the plasma kinetics of EPA and DHA over a 72‐h period following a single dose ingestion of EPA EE versus DHA EE concentrates. Since, post‐load plasma kinetic profiles of EPA and DHA after a single dose intake of highly purified unlabeled EPA and DHA products has not been studied, our study is of exploratory nature.

## MATERIALS AND METHODS

### Study design

This study adhered to the tenets of the Declaration of Helsinki. All protocols involving human participants were approved by the Ethics Committee of the Medical Association of Lower Saxony (Hanover, Germany). All participants provided signed informed consent. The study is registered in the German Clinical Trials Register (ref. DRKS00012257).

The study was conducted in the year 2018 at the Institute of Food and One Health, Leibniz University Hanover, Germany. The study was conducted in a cross‐over design with sequentially administration of EPA and DHA concentrates, which eliminates inter‐individual variability and ensures that each participant acts as their own control. Moreover, this study design provides benefits such as consistency in baseline conditions (e.g., metabolic rate, health status, and environmental factors), resulting in reduced data noise.

**FIGURE 1:**
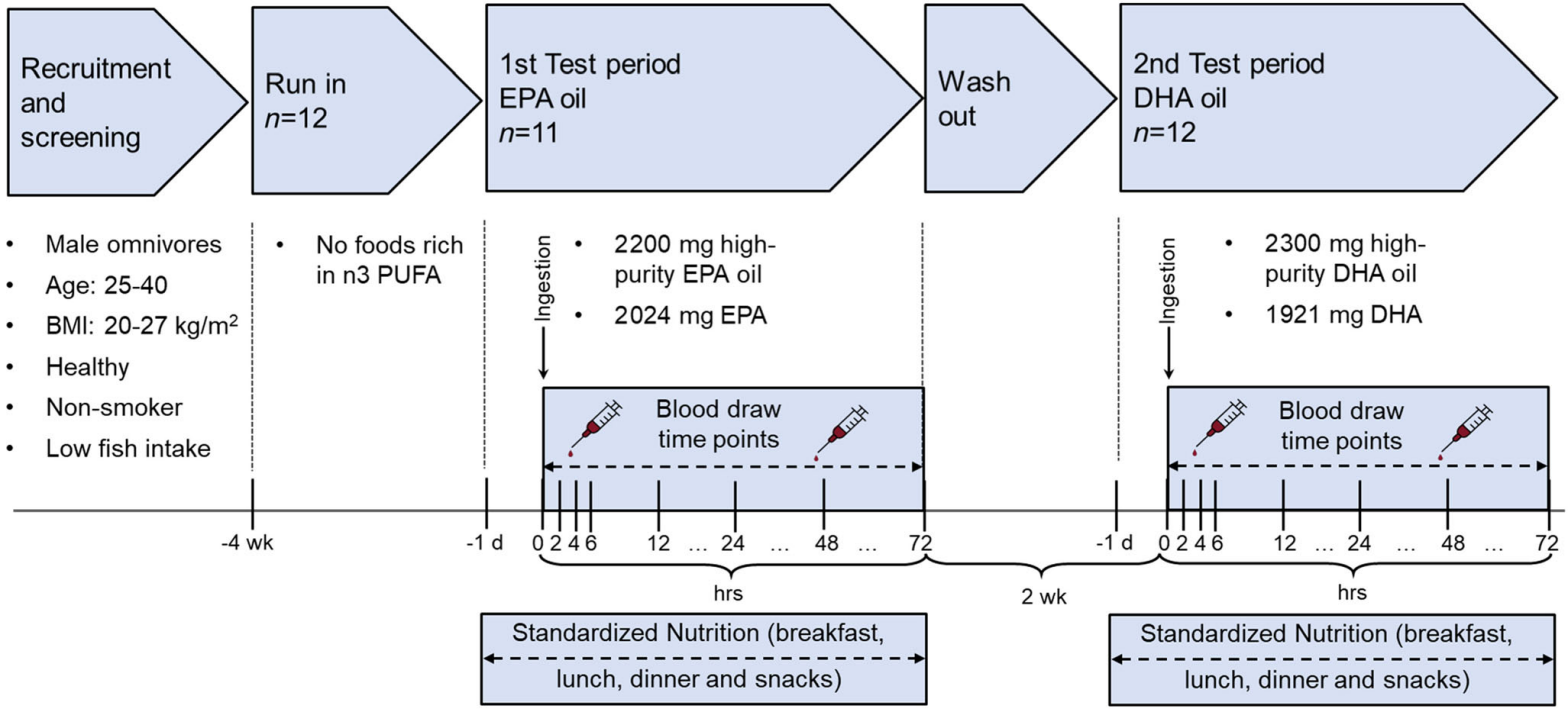
Schematic presentation of the study design and methods.

The study consisted of a recruitment‐screening phase, two 72‐h test periods separated by a 2‐week wash‐out period (Figure 1). A 4‐week run‐in phase was done to stabilize dietary habits. A 72‐h follow‐up period provides a balanced timeframe to obtain a representative kinetic profile as it is long enough to observe the absorption, distribution, metabolism, and initial excretion phases of n3 PUFAs. Extending the follow‐up to 4 or more days inevitably leads to lower subject compliance and may introduce variability due to external factors such as diet, lifestyle changes or other medications. After recruiting and screening, the pre‐selected participants underwent a screening examination.

### Participants

Participants were recruited from the general population of Hanover, Germany, through advertisements in newspapers. To minimize inter‐individual variability in plasma n3 PUFA levels, a homogeneous study population was selected, consisting solely of males to eliminate hormonal influences from females. Moreover, only individuals with low fish intake were recruited to ensure a consistent n3 PUFA baseline status. Screening questionnaires were used to pre‐qualify individuals based on specific inclusion criteria: “being male”, aged between 20 and 40 years, having a body mass index (BMI) between 20 and 27 kg/m^2^, and following a mixed diet with low fish consumption (≤1 times per week). Exclusion criteria were smoking, frequent consumption of fish (≥2 times per week), chronic diseases, and various drugs (for details see Data S1). Inclusion and exclusion criteria were assessed using questionnaires.

### Test oils

Test products were purchased from KD Pharma (Bioggio, Switzerland). In both test products, the fatty acids were provided in the EE form(Table 1).

**TABLE 1:** Fatty acid pattern of the EPA and DHA concentrates used in the study.

Fatty acid	Common name	%/total fatty acids	
		EPA	DHA
16:0	Palmitic acid	0.06	0.08
18:0	Stearic acid	0.03	0.03
18:4n3	Stearidonic acid	0.41	<LLOQ
20:4n6	Arachidonic acid	0.25	<LLOQ
20:4n3	Eicosatetraenoic acid	0.59	<LLOQ
20:5n3	Eicosapentaenoic acid	98.7	0.21
22:5n6	Docosapentaenoic acid n6	<LLOQ	0.74
22:5n3	Docosapentaenoic acid n3	<LLOQ	2.18
22:6n3	Docosahexaenoic acid	<LLOQ	96.7

*Note*: <LLOQ, below lower limit of quantification.

### Procedure

The procedure was exactly the same during both test periods. All measurements were carried out by trained nutritionists from the institute. Participants completed a questionnaire to collect data on changes in medication, diet, and lifestyle (such as physical activity) since the screening questionnaire.

Blood samples were taken before the test products were ingested (t0, baseline) and 2, 4, 6, 8, 12, 24, 48, and 72 h (t0‐t72) thereafter using Multifly needles (Sarstedt, Nümbrecht, Germany). Blood was collected into serum and EDTA collection tubes (Sarstedt). For the determination of plasma FA concentrations, the EDTA tubes were centrifuged at 1500 × *g* for 10 min at 4°C. The plasma was then transferred to 1.5 mL plastic containers (Sarstedt), immediately frozen, and stored at −80°C until further processing and analysis. All transfer procedures were performed while maintaining a cold environment with ice.

After first blood draw and anthropometric measurements, the concentrates (EPA in the first test period, DHA in the second test period) were administered. To enable more precise dosage, test products were given pure as a liquid on a spoon. 2.2 g of EPA EE was administered, resulting in a total of 2024 mg of EPA. Due to a slightly lower concentration of DHA in the DHA EE product compared to EPA in the EPA EE product, the amount of DHA EE administered was 2.3 g, resulting in a total of 1921 mg of DHA. The test products were given together with a standardized breakfast which consisted of a wheat roll with cream cheese, chicken breast cold cuts, Gouda cheese, strawberry jam, butter, a glass of orange juice, and milk (1.5% fat). The total fat amount in the breakfast was 36.6 g, equivalent to 330 kcal.

A standardized diet was provided to the participants to control especially the PUFA intake during the intervention period. The standardized meal plan started with lunch 1 day before the first blood draw and ended with the final blood sample 72 h later. The diet devoided EPA and DHA, and the portion sizes were customized according to the participants' energy requirements. Participants were free to consume water, tea, and coffee (without milk/sugar) ad libitum. The macronutrient and FA pattern of the standardized diet is described in detail in the Table S1.

Moreover, the participants were asked not to eat fish, seafood, alpha‐linolenic acid (aLNA, 18:3n3)‐rich oils, and foods over the entire study.

Participants were asked about side effects after taking the test products.

### Analysis of fatty acids in plasma

The plasma fatty acid levels were determined by gas chromatography (GC) with flame ionization detection (FID) as described (Ostermann et al., 2014) with minor modifications. Blood plasma lipids were extracted with methyl tert‐butyl ether and methanol to allow subsequent transesterification with hydrogen chloride in methanol. The resulting fatty acid methyl esters (FAMEs) were quantified with FAME 25:0 as the internal standard. Plasma levels were calculated as μg of fatty acid per milliliter of plasma.

### Mathematical and statistical analysis

If the plasma concentration of a fatty acid was below the lower limit of quantification (LLOQ) in a maximum of 50% of the samples, the LLOQ (e.g., 0.5 μg/mL) was inserted to calculate mean and SE for this respective fatty acid. If the plasma concentration of a fatty acid could not be quantified in more than 50% of the samples, the LLOQ was given instead of mean ± SE, and no statistical evaluation was performed for this parameter. Areas under the concentration curves (AUCs) for plasma EPA and DHA were calculated for each person during each intervention period according to the trapezoidal rule. To measure the net response over baseline, incremental AUC (iAUC) were calculated by subtracting the baseline area from the total AUC. Time (t_max_) to maximum concentrations (C_max_) were identified by visual inspection of the data.

Decay modeling was performed to determine the half‐life (t_1/2_) for plasma levels of EPA and DHA. Concentrations after C_max_ were first corrected for baseline concentrations. These logarithmized concentration data were plotted against time and a linear regression was performed. The slope of the line (k) represents the elimination rate constant. Half‐life was calculated using the formula: t_1/2 =_ $\frac{\ln\left(2\right)}{k}$.

iAUC for EPA and DHA were defined as the primary outcome parameters. All other markers were secondary. Statistical analyses were performed using SPSS software (IBM SPSS Statistics 28; Chicago, IL, USA). Due to the skewed distribution of some of the data, we used a non‐parametric approach. The non‐parametric Friedman test followed by Dunn‐Bonferroni post hoc test was used for further analysis. Statistical tests were only performed for fatty acids that were quantified in the study population at all time points. Statistical significance was set at *p* ≤ 0.05.

## RESULTS

### Study population

Twelve male volunteers (mean ± SD age: 24.6 ± 2.43 years) met the study criteria and were involved in the study population. Based on the parameters collected (Table 2) and self‐reports in the questionnaires, participants were considered to be generally healthy. The mean ± SD BMI was 24.6 ± 2.06 kg/m^2^. All participants stated that they normally eat an omnivorous diet with a low intake of fish. The participants stated that they had not consumed any fish or seafood during the entire study period. The test products were well tolerated, and no adverse side effects were reported. All participants completed the study (no dropouts). In the test period with the EPA EE, one sample had to be excluded from the study due to a technical error (clot formation after centrifugation).

**TABLE 2:** Plasma concentrations (μg/mL) of selected fatty acids and sum fatty acids at baseline (t0) and 2–72 h (t2–t72) after single‐dose ingestion of (A) EPA EE concentrate (first test period) and (B) DHA EE concentrate (second test period).

	t0		t2		t4		t6		t8		t24		t48		t72	
	mean ± SE		mean ± SE		mean ± SE		mean ± SE		mean ± SE		mean ± SE		mean ± SE		mean ± SE	
**(A) First test period (*n* = 11)**																
20:5n3	22.3 **±** 2.76		26.3 **±** 2.61		49.8 **±** 9.99		105.9 **±** 13.3		66.4 **±** 7.22		64.6 **±** 4.52		52.4 **±** 4.76		40.9 **±** 3.78	
22:5n3	23.7 **±** 1.90		23.0 **±** 1.35		24.2 **±** 1.88		25.3 **±** 1.41		23.1 **±** 1.99		28.2 **±** 1.86		28.5 **±** 2.53		29.6 **±** 2.19	
22:6n3	49.9 **±** 5.74		47.2 **±** 4.48		47.9 **±** 5.14		47.4 **±** 4.43		45.8 **±** 5.11		48.9 **±** 4.93		47.3 **±** 4.92		48.5 **±** 4.48	
∑FA	3170 **±** 200		3558 **±** 294	n.s.	3706 **±** 321	n.s.	3985 **±** 329	**	3213 **±** 313	n.s.	2835 **±** 250	n.s.	2834 **±** 263	n.s.	2892 **±** 244	n.s.
∑SFA	853 **±** 67.7		1090 **±** 120	*	1184 **±** 136	**	1293 **±** 151	***	1007 **±** 132	n.s.	772 **±** 77.8	n.s.	792 **±** 91.3	n.s.	821 **±** 87.7	n.s.
∑MUFA	997 **±** 74.1		1122 **±** 102	n.s.	1129 **±** 98.1	n.s.	1189 **±** 99.7	n.s.	886 **±** 91.6	n.s.	813 **±** 77.0	*	760 **±** 62.8	*	803 **±** 71.8	*
∑PUFA	1320 **±** 85.7		1346 **±** 92.3	n.s.	1393 **±** 107	n.s.	1503 **±** 99.6	*	1320 **±** 108	n.s.	1250 **±** 105	n.s.	1288 **±** 117	n.s.	1268 **±** 95.6	n.s.
∑n3 PUFA	120 **±** 10.6		122 **±** 7.44	n.s.	147 **±** 11.4	n.s.	206 **±** 18.7	***	155 **±** 14.1	*	161 **±** 11.8	**	147 **±** 12.3	n.s.	141 **±** 11.1	n.s.
∑n6 PUFA	1200 **±** 79.1		1224 **±** 86.9	n.s.	1245 **±** 99.0	n.s.	1298 **±** 87.2	n.s.	1165 **±** 96.4	n.s.	1089 **±** 94.5	n.s.	1141 **±** 106	n.s.	1127 **±** 85.8	n.s.
**(B) Second test period (*n* = 12)**																
20:5n3	17.5 **±** 1.98		16.8 **±** 2.01		16.7 **±** 1.80		17.3 **±** 2.08		17.5 **±** 2.10		17.7 **±** 1.70		16.6 **±** 1.61		16.8 **±** 1.83	
22:5n3	17.4 **±** 1.63		17.8 **±** 1.60		17.5 **±** 1.47		18.4 **±** 1.69		17.4 **±** 1.49		17.3 **±** 1.48		16.0 **±** 1.38		16.4 **±** 1.48	
22:6n3	37.9 **±** 4.25		41.4 **±** 3.84		46.2 **±** 5.66		80.0 **±** 12.6		58.0 **±** 6.84		51.3 **±** 3.67		49.7 **±** 4.39		51.7 **±** 5.38	
∑FA	2973 **±** 256		3210 **±** 278	n.s.	3322 **±** 264	*	3449 **±** 346	n.s.	3067 **±** 272	n.s.	2817 **±** 162	n.s.	2728 **±** 195	n.s.	2839 **±** 234	n.s.
∑SFA	1002 **±** 99.4		1181 **±** 120	*	1255 **±** 121	**	1327 **±** 162	***	1147 **±** 131	n.s.	974 **±** 66.8	n.s.	936 **±** 74.4	n.s.	993 **±** 86.0	n.s.
∑MUFA	879 **±** 89.8		930 **±** 95.9	n.s.	954 **±** 91.0	n.s.	957 **±** 109	n.s.	808 **±** 88.0	n.s.	771 **±** 55.9	n.s.	708 **±** 51.2	**	752 **±** 68.8	n.s.
∑PUFA	1092 **±** 79.3		1099 **±** 76.2	n.s.	1113 **±** 63.4	n.s.	1164 **±** 85.7	n.s.	1113 **±** 60.5	n.s.	1072 **±** 50.2	n.s.	1085 **±** 74.9	n.s.	1095 **±** 82.9	n.s.
∑n3 PUFA	88.7 **±** 8.84		94.0 **±** 8.21	n.s.	98.3 **±** 9.48	n.s.	134 **±** 16.4	***	108 **±** 10.4	*	102 **±** 7.03	*	97.0 **±** 8.04	n.s.	103 **±** 9.82	*
∑n6 PUFA	1003 **±** 71.4		1005 **±** 69.0	n.s.	1015 **±** 55.6	n.s.	1031 **±** 71.9	n.s.	1005 **±** 52.7	n.s.	969 **±** 44.4	n.s.	988 **±** 67.4	n.s.	992 **±** 73.5	n.s.

*Note*: All results are expressed as mean ± SE. n.s., not significant compared with t0.

*** *p* < 0.001.

** *p* ≤ 0.005.

* *p* ≤ 0.05.

### Plasma FA concentrations

**FIGURE 2:**
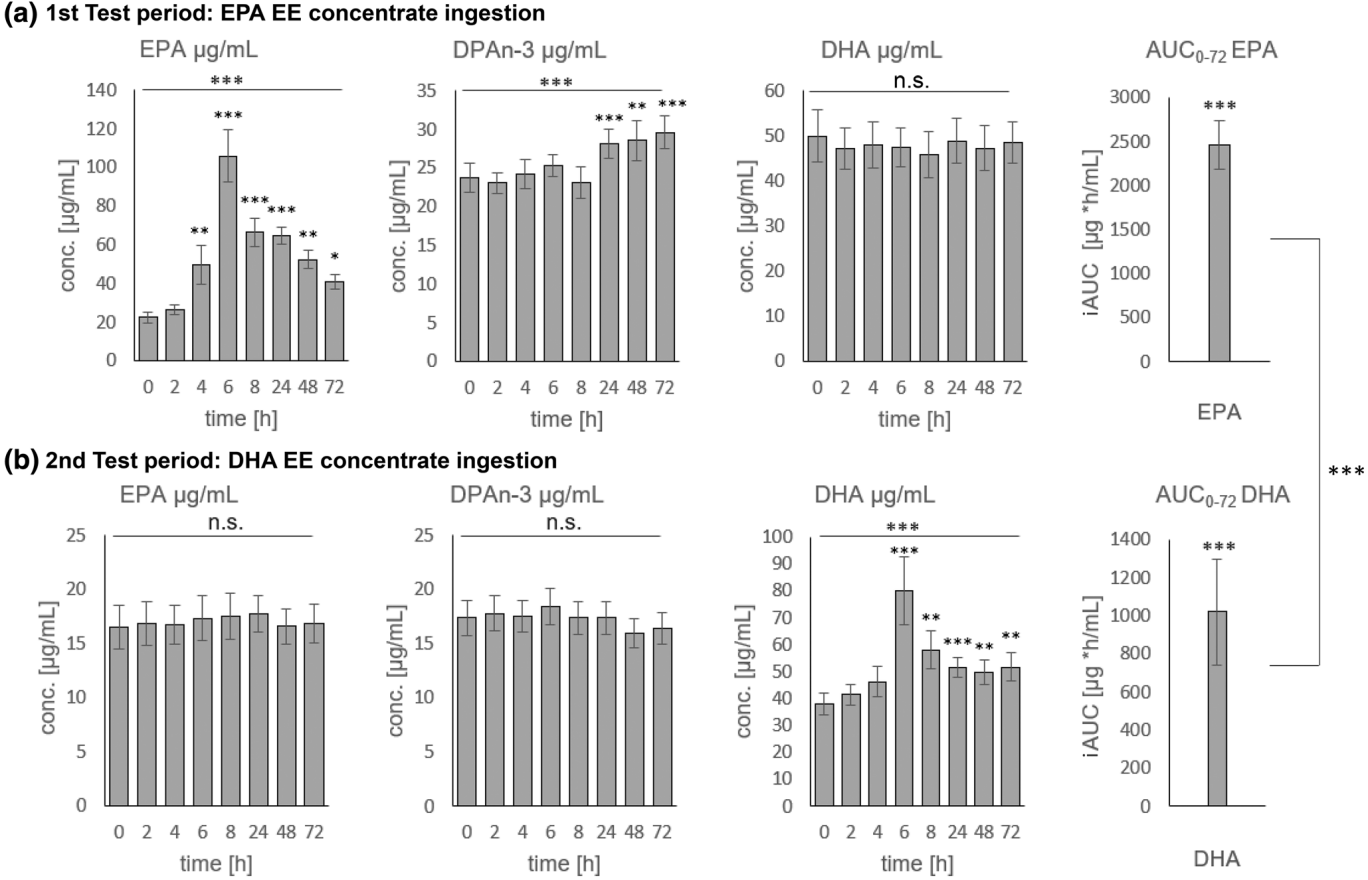
Plasma concentration of EPA, DPAn3, and DHA at baseline and 2 to 72 h after single‐dose ingestion of (a) EPA EE (2.2 g) and (b) DHA EE (2.3 g) concentrates. All results are expressed as mean ± SE. ***p < 0.001; **p ≤ 0.005; *p ≤ 0.05; n.s., not significant; iAUC, incremental Area under the curve.

Figure 2 shows the course of plasma concentrations of EPA, DPAn3, and DHA after administration of the test concentrates. The complete FA patterns are shown in Tables S2 and S3. Baseline levels of EPA were 4.9 ± 2.1 μg/mL (*p* = 0.04) higher at the beginning of first study period than in second test period. For DHA the difference was 11.0 ± 9.5 μg/mL (*p* = 0.003).

Only 4 h after the administration of EPA, the plasma EPA level had more than doubled. After 6 h, the plasma EPA level peaked at more than 4.7 times the baseline level. At the subsequent time points (8, 24, 48, and 72 h), plasma EPA levels decreased linearly but were still 3.0, 2.9, 2.3, and 1.8 times higher than baseline. In addition, a significant increase in plasma DPAn3 concentrations was observed 24 h after administration of EPA EE, while no change was visible at earlier time‐points. From this point on, the DPAn3 plasma concentrations also increased linearly. No changes in DHA plasma concentrations were observed at any time. Despite DHA plasma concentrations following DHA EE administration showing a similar trend to that of EPA after EPA EE intake, the overall rise in concentrations was noticeably lower. Six hours post‐administration, plasma DHA levels were merely 2.1 times higher than the baseline. Following the peak after 6 h, plasma DHA concentrations ranged between 1.3 and 1.5 times higher compared with baseline. The mean (±SE) C_max_ values for EPA and DHA were 115 ± 11 μg/mL and 86 ± 11 μg/mL (*p* = 0.05), respectively. The mean (±SE) t_max_ values for EPA was 7.3 ± 1.7 h compared with DHA at 5.8 ± 0.4 h. Furthermore, the mean ± SE iAUC_0‐72_ for EPA (2461 ± 279 μg/mL) significantly (*p* < 0.001) exceeded that for DHA (1021 ± 170 μg/mL), corresponding to a 2.4 times higher iAUC_0‐72_ value. No relevant changes in concentrations of all other PUFA—including EPA and DPAn3 were observed after DHA EE administration. Particularly due to the strong increase in EPA and DHA levels in plasma after EPA and DHA EE ingestion, respectively, the Σn3 PUFA levels also increased significantly (at t6, t8, and t24) with both EPA and DHA and t72 in addition for DHA. EPA EE administration led to a significantly greater increase in the concentration of total PUFA compared to DHA EE administration.

The median half‐life of EPA was 30 h, with a range of 26 to 76 h (25th and 75th percentiles, respectively). For DHA, the median half‐life was 88 h, with a range of 31 to 107 h. The mean ± SE half‐life was for EPA and DHA was 45 ± 8 and 66 ± 12 h (n.s.), respectively.

## DISCUSSION

While high‐purity EPA and DHA supplements are accessible and utilized in clinical studies, there is still a lack of comprehensive understanding regarding the absorption kinetics of EPA and DHA from these oils. We observed that the mean area under the plasma concentration curve over 72 h for EPA after EPA EE concentrate ingestion was higher than that for DHA after DHA EE concentrate ingestion.

Investigating the kinetics of EPA and DHA in human studies with unlabeled FAs is challenging. Various factors such as age (Flock et al., 2013; Gellert et al., 2017; Harris et al., 2012), sex (Bakewell et al., 2006; Crowe et al., 2008; Metherel et al., 2009), BMI (Flock et al., 2013; Langlois & Ratnayake, 2015; Sands et al., 2005), dietary components (Crowe et al., 2008; Flock et al., 2013; Sala‐Vila et al., 2011) and smoking (Harris et al., 2012; Langlois & Ratnayake, 2015) influence the metabolism of PUFA and impact the intra‐ and inter‐individual variability of blood n3 PUFA levels (Groot et al., 2019). To minimize this variability, a homogeneous group of healthy, non‐smoking men within a narrow range of age and BMI was selected to study the effect of a single dose of EPA versus DHA concentrate on plasma n3 PUFA concentrations. Furthermore, the study aimed to achieve a homogeneous study population by specifically including omnivores with low fish consumption. To avoid bias from the intake of PUFA—particularly n3 PUFA—from each participant's background diet, the participants were maintained on a standardized diet throughout the study phases, including two meals the day before the actual start of the study. The numerous—sometimes elaborate—methodological measures to control background fluctuations in n3 PUFA levels proved to be effective. The intra‐ and inter‐individual variability in plasma EPA and DHA levels, was significantly lower than in studies lacking these measures (Abdelmagid et al., 2015; Finnegan et al., 2003). Furthermore, we found no carry‐over effect from the EPA administration in the first study period to the second. On the contrary, n3 PUFA blood concentrations were even lower in the second period than in the first, probably due to the complete avoidance of fish and seafood throughout the study period.

Although the general course of the plasma concentrations of EPA and DHA is similar, our results show that plasma EPA levels increase significantly more than DHA levels when EPA or DHA concentrates are consumed at similar concentrations. This is evident from the significantly higher iAUC_0‐72_ values for EPA compared to DHA. Furthermore, the rise in plasma levels of sum n3 PUFA was significantly greater following the intake of EPA than DHA.

It is difficult to compare our data with the literature because there are no studies with a comparable design. Some studies have compared physiological effects of EPA vs DHA oils over a multiple‐dose intervention period of 10 weeks (Allaire et al., 2017) and 12 weeks (Klingel et al., 2019). A recent cross‐over study over 6 days of supplementation similarly found that EPA ingestion was followed by an effective integration of EPA into plasma lipid fractions, while DHA incorporation was restricted (Guo et al., 2020). Only a few single‐dose kinetic studies have been performed, but these have used n3 PUFA oils containing mixtures of EPA and DHA in varying proportions (Kang et al., 2023; Schneider et al., 2011; Schuchardt et al., 2011). Our results align with previous studies showing a quicker rise in several blood biomarkers of EPA compared to DHA following a single dose of EPA + DHA mixtures (Chevalier et al., 2021; Metherel et al., 2009; Schneider et al., 2011; Schuchardt et al., 2011). EPA also showed a curve with a peak at 8 h and then declined. The kinetic course of DHA, on the other hand, consisted of two phases. After an early increase, the levels fell quickly to almost the initial value, then increased again after 6 to 8 h. Similar observations in the comparison of EPA and DHA kinetic profiles have been made in previous studies (Chevalier et al., 2021; Metherel et al., 2009; Schneider et al., 2011), although in these studies EPA + DHA mixtures were administered. For both EPA and DHA, plasma concentrations are still significantly above baseline concentrations after 72 h. The mean half‐life shows that DHA takes longer to return to baseline plasma levels, at almost 66 h, than EPA, at around 45 h. There is considerable inter‐individual variability in the course of the post‐peak elimination phase. The range of half‐lives is from 13 to 110 h for DHA and from 12 to 94 h for EPA.

In a randomized, open‐label cross‐over trial, Kang et al. (2023) compared the bioavailability of EPA versus DHA in liquid crystalline nanoparticle‐based formulations (Kang et al., 2023). 50 healthy male participants ingested a single‐dose of EE concentrates as either 1840 mg EPA or 1520 mg DHA. Blood samples were taken over 72 h at 8 time points. The concentrations of EPA in plasma peaked after 6 h, then decreased, only to rise again after 24 h. Even after 72 h, the concentrations remained above baseline. DHA concentrations peaked also at 6 h but decreased more slowly. Similarly, baseline was still not reached after 72 h. Although the kinetics of EPA and DHA are different from our results, there are some similarities. Similar to our findings, Kang et al. (2023) observed a greater increase for plasma EPA (AUC_0−72_ 930 ± 285 μg‧h/mL) compared with DHA (541 ± 287 μg h/mL). However, it should be noted that the dose of EPA administered was significantly higher than the dose of DHA in the study by Kang et al.

The reasons for the different course of EPA and DHA cannot be clearly clarified. It is rather unlikely that higher EPA plasma levels compared with DHA are the result of a greater gastrointestinal absorption (cleavage, uptake) of EPA EE compared to DHA EE. The level of EPA and DHA in plasma reflect the balance between uptake and release of fatty acids from organs and tissues and thus, it is also possible that DHA is removed more quickly from the circulation and enters the different tissues at a higher rate than EPA hence supporting different roles for EPA and DHA (Klingel et al., 2019). Polozova and Salem observed that labeled ^14^C‐DHA distributes differently compared to ^3^H‐oleic acid after intravenous co‐injection in mice (Polozova & Salem, 2007). The authors found that ^14^C‐DHA preferentially distributes to liver and concluded that DHA is specifically taken up by liver, esterified, loaded into lipoproteins, and then delivered to brain, heart, and other target tissues. Perhaps EPA is more evenly distributed. Another theory is that DHA is used directly as a substrate for β‐oxidation in peroxisomes and is thus used for energy (Plourde et al., 2014). It is important to note that the results of single‐dose studies do not indicate the long‐term availability of EPA and DHA. For example, a multiple‐dose study over 10 weeks found that DHA supplementation increased the Omega‐3 index significantly more than an equivalent amount of EPA (Allaire et al., 2017).

EPA and DHA baseline concentrations were lower in the second test period (DHA) compared to the first (EPA). The reason for these differences is unknown, but the somewhat longer period of assiduously avoiding fish consumption may have played a role. Although these differences were statistically significant, they were unlikely to be physiologically relevant. The baseline EPA and DHA concentrations at both time points are still within a normal range reported in Germany and in other Western countries (Braeckman et al., 2014; Metherel et al., 2019; Plourde et al., 2014; Schuchardt et al., 2016). Although the variations remain within the range of typical physiological fluctuations, these differences might influence the results. A recent study has shown that DHA inhibits EPA elongation (Metherel et al., 2024). Higher DHA levels at baseline during EPA EE administration could have a significant effect on EPA elongation and could explain, at least in part, the higher EPA levels after EPA EE administration (resulting from lower EPA elongation efficiency) compared with the DHA dose‐related response. In this context, the absence of randomization is to be regarded as a limitation. Due to the small concentration differences between EPA and DHA in the test products, 100 mg less EPA EE was administered than DHA EE. However, the small difference probably only contributes slightly to the significantly lower post‐dosing plasma DHA levels compared to EPA.

In addition to the differences between EPA and DHA, changes in plasma concentrations of other n3 PUFA were also observed. We found an increase in plasma DPAn3 concentrations after 24 h of EPA concentrate intake, suggesting a conversion to DPAn3. On the other hand, we could not observe a change in DHA concentrations after EPA administration. Consistent with our findings, Léveillé et al. (2017) also observed no increase in circulating DHA levels in the first 24 h after EPA administration (Léveillé et al., 2017). However, the lack of increase in plasma DHA levels with EPA administration does not mean that no conversion of EPA to DHA occurred. Rather, it indicates that regardless of how much EPA was converted to DHA, it was associated with a similar rate of metabolic consumption of DHA (Metherel et al., 2019).

### Strength and limitations

This study demonstrates strengths in terms of the clear and straight‐forward design including participant selection to ensure a homogenous cohort and tightly controlled conditions during the test phase. However, this study has some limitations, including the small sample size and the lack of a control group. As discussed above, the absence of randomization might also be viewed as a limitation. The omission of women reduces generalizability of the findings. A comparison group of women with otherwise identical inclusion and exclusion criteria would have been useful. Despite dosing the concentrates by weighing, there were slight differences in the administered amount of EPA and DHA between the two test periods. The use of unlabeled EPA and DHA precluded a true exploration of kinetics (which would require tracking labeled FAs) hence conclusions regarding absorption efficiency should be considered tentative. The original study design was not designed to calculate plasma half‐life of FA. C_max_ was 6 or 8 h for most subjects. With only a few measurement points (8, 24, 48, and 72 h) a robust model for the concentration decay was not possible.

## CONCLUSION

Our findings highlight the distinct plasma kinetics of EPA and DHA. The results indicate that administration of EPA EE increased post‐dosing plasma EPA levels to a greater extent than administration of DHA EE affects plasma DHA levels, suggesting greater systemic availability of EPA. Animal tracer studies are needed to determine the metabolic reasons for observed differences and if EPA and DHA distribute differently in the body after uptake.

## AUTHOR CONTRIBUTIONS

**HMLS:** Investigation; formal analysis; data curation; writing**—**original draft; visualization. **TG:** Investigation; formal analysis. **IS:** Formal analysis; **SW** and **VC:** Investigation; data curation; validation; investigation. **LK:** Investigation; data curation; validation; writing**—**review and editing. **AH:** Resources; writing—review and editing. **WSH:** Writing—review and editing. **NHS:** Resources; conceptualization; methodology; funding acquisition; supervision; writing—review and editing. **JPS:** Conceptualization; methodology; funding acquisition; supervision; writing—review and editing; project administration.

## FUNDING INFORMATION

This study was supported by a Grant of the German Research Foundation to NHS (SCHE 1801) and JPS (SCHU 2516).

## CONFLICT OF INTEREST STATEMENT

The authors declare that they have no conflict of interest.

## ETHICS STATEMENT

The study received ethical approval from the Ethics Committee of the Medical Association of Lower Saxony (Hannover, Germany) and written informed consent was obtained from all study participants. The assessment and processing of the data were completed following the Lower Saxony Data Protection Act, adhering to the Declaration of Helsinki and the principles of Good Clinical Practice.

## Supporting information


**Data S1.** Supporting Information.

**Data S2.** Supporting Information.
