# Supplementary material for: Plasma levels of EPA and DHA after ingestion of a single dose of EPA and DHA ethyl esters
Source: Lipids. 2024 Sep 19;60(1):15–23. doi: 10.1002/lipd.12417 (PMC11717491; doi:10.1002/lipd.12417)
Supplement: Supplementary file 1 — Data S1. Supporting Information. [file LIPD-60-15-s001.docx]

**Supplementary information**

Exclusion criteria

- Being female
- < 20 and > 40 years of age
- BMI < 20 and > 27 kg/m2
- Smoking
- Frequent consumption of fish (>2 times per week)
- Dependence on alcohol, drugs or medication
- Serum triglyceride (TG) levels ≥150 mg/dl (≥1.7 mmol/l)
- Serum total cholesterol levels ≥200 mg/dl (≥5.2 mmol/l)
- Intake of fish (>2 times per week)
- Chronic diseases (e.g. malignant tumors, manifest cardiovascular disease, insulin-dependent type 1 and 2 diabetes, severe renal or liver diseases); chronic gastrointestinal disorders (especially small intestine, pancreas, liver) as well as prior gastrointestinal surgical procedures (e.g. gastrectomy); hormonal disorders (e.g. Cushing ́s syndrome and untreated hyperthyroidism); uncontrolled hypertension; blood coagulation disorders
- Intake of coagulation-inhibiting drugs; periodic intake of laxatives; intake of anti-inflammatory drugs (incl. acetylsalicylic acid); intake of lipid lowering drugs or supplements during the last 3 months before baseline examination
