# Supplementary material for: Plasma levels of EPA and DHA after ingestion of a single dose of EPA and DHA ethyl esters
Source: Lipids. 2024 Sep 19;60(1):15–23. doi: 10.1002/lipd.12417 (PMC11717491; doi:10.1002/lipd.12417)
Supplement: Supplementary file 2 — Data S2. Supporting Information. [file LIPD-60-15-s002.docx]

# **Supplementary Tables and Figures**

**Table S1:** Daily energy, macronutrient, and fatty acid intake of study participants during the whole period of the standardized nutrition **(A)** and energy, macronutrient and fatty acid intake of meals at Day 1 of the standardized nutrition **(B)**.

| **A)** | **Day 1** | | **Day 2** | | **Day 3** | |
| --- | --- | --- | --- | --- | --- | --- |
| **Portion size** | **small** | **large** | **small** | **large** | **small** | **large** |
| **Energy intake (kcal)^a^** | 2,924 | 3,152 | 2,687 | 2,946 | 2,907 | 3,179 |
| **Carbohydrates (g)^a^** | 337 | 378 | 321 | 375 | 335 | 375 |
| **Protein (g)^a^** | 122 | 128 | 103 | 110 | 125 | 136 |
| **Total fat intake (g)^b^** | 82.0 | 113 | 102 | 103 | 108 | 85.3 |
| **SFA (g)^b^** | 37.1 | 35.3 | 40.9 | 38.6 | 38.5 | 37.3 |
| **MUFA (g)^b^** | 17.5 | 16.6 | 20.9 | 20.1 | 18.1 | 17.7 |
| **PUFA (g)^b^** | 3.98 | 4.00 | 10.2 | 10.2 | 4.86 | 5.32 |
| **LA (g)^b^** | 3.25 | 3.27 | 9.49 | 9.45 | 4.00 | 4.36 |
| **aLNA (g)^b^** | 0.53 | 0.52 | 0.51 | 0.49 | 0.67 | 0.74 |
| **ARA (g)^b^** | 0.10 | 0.11 | 0.12 | 0.12 | 0.11 | 0.11 |
| **EPA (g)^b^** | 0.03 | 0.03 | 0.03 | 0.03 | 0.03 | 0.03 |
| **DPAn3 (g)^b^** | <0.01 | 0.02 | <0.01 | 0.02 | 0.01 | 0.02 |
| **DHA (g)^b^** | <0.01 | <0.01 | <0.01 | <0.01 | <0.01 | <0.01 |

| **B)** | **Breakfast** | **Lunch** | **Snack** | | **Dinner** | |
| --- | --- | --- | --- | --- | --- | --- |
| **Portion size** | **small / large** | **small / large** | **small** | **large** | **small** | **large** |
| **Energy intake (kcal)^a^ (kcal)^a^** | 900 | \| 919 \| 863.39 \| 222.5 \| 319.08 \| 881.76 \| 1013.92 \| \| --- \| --- \| --- \| --- \| --- \| --- \|   687  919   \| 919.3 \| 863.39 \| 222.5 \| 319.08 \| 881.76 \| 1013.92 \| \| --- \| --- \| --- \| --- \| --- \| --- \|   687 | 223 | 319 | 882 | 1,014 |
| **Carbohydrates (g)^a^** | 95.9 | 125 | 33.3 | 48.1 | 83.3 | 109 |
| **Protein (g)^a^** | 34.8 | 37.7 | 8.00 | 10.0 | 41.2 | 45.3 |
| **Total fat intake (g)^b^** | 36.6 | 6.80 | 3.78 | 4.10 | 34.9 | 35.1 |
| **SFA (g)^b^** | 17.82 | 2.71 | 2.16 | | 14.4 | 12.6 |
| **MUFA (g)^b^** | 8.67 | 1.24 | 1.07 | | 6.49 | 5.64 |
| **PUFA (g)^b^** | 1.86 | 0.33 | 0.12 | | 1.67 | 1.69 |
| **LA (g)^b^** | 1.54 | 0.24 | 0.09 | | 1.38 | 1.40 |
| **aLNA (g)^b^** | 0.22 | 0.07 | 0.02 | | 0.21 | 0.20 |
| **ARA (g)^b^** | 0.05 | 0.01 | <0.01 | | 0.04 | 0.04 |
| **EPA (g)^b^** | 0.02 | <0.01 | <0.01 | | 0.01 | 0.01 |
| **DPAn3 (g)^b^** | <0.01 | <0.01 | <0.01 | | <0.01 | 0.01 |
| **DHA (g)^b^** | <0.01 | <0.01 | <0.01 | | <0.01 | <0.01 |

*Levels are shown at day 1, 2 and 3 of standardized nutrition for small and large portion size.*

*ARA, arachidonic acid; aLNA, α-linolenic acid; EPA, eicosapentaenoic acid; DHA, docosahexaenoic acid; DPAn3, docosapentaenoic acid; LA, linoleic acid; MUFA, monounsaturated fatty acids; PUFA, polyunsaturated fatty acids; SFA, saturated fatty acids.*

*^a^ Calculated with PRODI®*

*^b^ Calculated from own analyses.*

| **Table S2:** Full plasma fatty acid concentrations [µg/mL] after single-dose ingestion of the EPA concentrate. All results are expressed as mean ± SE (n = 11). | | | | | | | | | | | | | | | | | | | | | | | | | |
| --- | --- | --- | --- | --- | --- | --- | --- | --- | --- | --- | --- | --- | --- | --- | --- | --- | --- | --- | --- | --- | --- | --- | --- | --- | --- |
|  | | | |  |  |  |  |  |  |  |  |  |  |  |  |  |  |  |  |  |  |  |  |  | |
|  | **t0** |  |  | **t2** |  |  | **t4** |  |  | **t6** |  |  | **t8** |  |  | **t24** |  |  | **t48** |  |  | **t72** |  |  | |
| **fatty acid** | **mean** | **±** | **SE** | **mean** | **±** | **SE** | **mean** | **±** | **SE** | **mean** | **±** | **SE** | **mean** | **±** | **SE** | **mean** | **±** | **SE** | **mean** | **±** | **SE** | **mean** | **±** | **SE** | |
| **10:0** | <LLOQ |  |  | <LLOQ |  |  | <LLOQ |  |  | <LLOQ |  |  | <LLOQ |  |  | <LLOQ |  |  | <LLOQ |  |  | <LLOQ |  |  |  |
| **11:0** | <LLOQ |  |  | <LLOQ |  |  | <LLOQ |  |  | <LLOQ |  |  | <LLOQ |  |  | <LLOQ |  |  | <LLOQ |  |  | <LLOQ |  |  |  |
| **12:0** | <LLOQ |  |  | <LLOQ |  |  | <LLOQ |  |  | <LLOQ |  |  | <LLOQ |  |  | <LLOQ |  |  | <LLOQ |  |  | <LLOQ |  |  |  |
| **13:0** | <LLOQ |  |  | <LLOQ |  |  | <LLOQ |  |  | <LLOQ |  |  | <LLOQ |  |  | <LLOQ |  |  | <LLOQ |  |  | <LLOQ |  |  |  |
| **14:0** | 3.94 | ± | 1.09 | 7.44 | ± | 2.79 | 12.3 | ± | 4.55 | 17.1 | ± | 6.33 | 8.90 | ± | 3.56 | 2.13 | ± | 0.35 | 4.78 | ± | 1.88 | 4.74 | ± | 2.01 | |
| **14:1n5** | 0.00 | ± | 0.00 | 0.00 | ± | 0.00 | 0.00 | ± | 0.00 | 0.00 | ± | 0.00 | 0.00 | ± | 0.00 | 0.00 | ± | 0.00 | 0.00 | ± | 0.00 | 0.00 | ± | 0.00 | |
| **15:0** | 3.25 | ± | 0.55 | 5.11 | ± | 0.98 | 6.28 | ± | 1.20 | 7.42 | ± | 1.63 | 4.83 | ± | 1.12 | 2.58 | ± | 0.46 | 3.13 | ± | 0.69 | 3.7 | ± | 0.79 | |
| **15:1n5** | <LLOQ |  |  | <LLOQ |  |  | <LLOQ |  |  | <LLOQ |  |  | <LLOQ |  |  | <LLOQ |  |  | <LLOQ |  |  | <LLOQ |  |  | |
| **16:0** | 523 | ± | 55.3 | 702 | ± | 89.8 | 768 | ± | 101 | 840 | ± | 113 | 636 | ± | 98.5 | 453 | ± | 56.6 | 469 | ± | 67.2 | 503 | ± | 67.7 | |
| **16:1n7** | 38.7 | ± | 6.06 | 50.9 | ± | 7.83 | 53.9 | ± | 8.40 | 56.4 | ± | 8.82 | 41.2 | ± | 7.91 | 32.7 | ± | 6.54 | 32.4 | ± | 6.01 | 37.8 | ± | 6.87 | |
| **17:0** | 9.02 | ± | 0.99 | 12.3 | ± | 1.45 | 13.7 | ± | 1.78 | 15.1 | ± | 1.76 | 11.9 | ± | 1.75 | 9.12 | ± | 1.09 | 9.04 | ± | 1.17 | 9.56 | ± | 1.10 | |
| **17:1n7** | <LLOQ |  |  | <LLOQ |  |  | <LLOQ |  |  | <LLOQ |  |  | <LLOQ |  |  | <LLOQ |  |  | <LLOQ |  |  | <LLOQ |  |  | |
| **18:0** | 263 | ± | 17.4 | 314 | ± | 26.6 | 334 | ± | 29.0 | 363 | ± | 31.4 | 297 | ± | 28.6 | 254 | ± | 17.9 | 254 | ± | 22.2 | 249 | ± | 19.6 | |
| **18:1n9** | 824 | ± | 62.2 | 931 | ± | 86.5 | 934 | ± | 84.2 | 985 | ± | 86.0 | 723 | ± | 77.5 | 657 | ± | 63.8 | 608 | ± | 49.9 | 643 | ± | 59.4 | |
| **18:1n7** | 74.5 | ± | 6.50 | 86.5 | ± | 8.30 | 87.3 | ± | 7.51 | 93.3 | ± | 6.61 | 68.7 | ± | 5.81 | 66.8 | ± | 5.95 | 63.4 | ± | 5.28 | 67.7 | ± | 5.41 | |
| **18:2n6** | 841 | ± | 62.0 | 872 | ± | 66.5 | 882 | ± | 73.4 | 926 | ± | 69.4 | 808 | ± | 69.8 | 734 | ± | 69.0 | 787 | ± | 76.9 | 777 | ± | 62.9 | |
| **18:3n6** | 13.1 | ± | 1.74 | 14.8 | ± | 2.15 | 15.0 | ± | 2.49 | 15.7 | ± | 2.39 | 13.2 | ± | 2.67 | 11.1 | ± | 1.66 | 10.8 | ± | 1.58 | 10.4 | ± | 1.76 | |
| **19:0** | 1.57 | ± | 0.26 | 2.23 | ± | 0.30 | 2.16 | ± | 0.24 | 2.24 | ± | 0.21 | 1.86 | ± | 0.23 | 1.49 | ± | 0.16 | 1.48 | ± | 0.19 | 1.56 | ± | 0.18 | |
| **18:3n3** | 18.6 | ± | 1.72 | 20.6 | ± | 2.01 | 20.2 | ± | 1.68 | 21.3 | ± | 1.81 | 15.2 | ± | 1.47 | 15.1 | ± | 1.59 | 14.5 | ± | 1.35 | 17.5 | ± | 1.70 | |
| **18:4n3** | 1.11 | ± | 0.14 | 1.34 | ± | 0.17 | 1.52 | ± | 0.25 | 1.67 | ± | 0.23 | 1.27 | ± | 0.27 | 1.22 | ± | 0.18 | 0.80 | ± | 0.13 | 0.91 | ± | 0.13 | |
| **20:0** | 7.66 | ± | 0.57 | 7.94 | ± | 0.39 | 8.16 | ± | 0.59 | 8.39 | ± | 0.63 | 7.57 | ± | 0.59 | 7.73 | ± | 0.54 | 7.62 | ± | 0.65 | 7.55 | ± | 0.54 | |
| **20:1n9** | 7.79 | ± | 0.81 | 7.50 | ± | 0.93 | 7.34 | ± | 0.61 | 7.54 | ± | 0.73 | 5.92 | ± | 0.45 | 5.93 | ± | 0.49 | 5.58 | ± | 0.56 | 5.61 | ± | 0.49 | |
| **20:2n6** | 8.44 | ± | 1.00 | 8.39 | ± | 0.91 | 8.12 | ± | 0.68 | 8.31 | ± | 0.69 | 7.03 | ± | 0.52 | 7.43 | ± | 0.68 | 7.42 | ± | 0.70 | 7.26 | ± | 0.53 | |
| **20:3n9** | 7.15 | ± | 1.13 | 7.05 | ± | 0.75 | 7.30 | ± | 0.80 | 7.36 | ± | 0.52 | 6.70 | ± | 0.56 | 6.49 | ± | 0.50 | 6.14 | ± | 0.53 | 5.93 | ± | 0.59 | |
| **20:3n6** | 64.9 | ± | 5.12 | 64.8 | ± | 5.33 | 65.5 | ± | 5.13 | 66.9 | ± | 3.86 | 63.8 | ± | 4.96 | 64.7 | ± | 4.26 | 64.2 | ± | 5.60 | 64.6 | ± | 4.83 | |
| **21:0** | <LLOQ |  |  | <LLOQ |  |  | <LLOQ |  |  | <LLOQ |  |  | <LLOQ |  |  | <LLOQ |  |  | <LLOQ |  |  | <LLOQ |  |  | |
| **20:4n6** | 255 | ± | 23.1 | 248 | ± | 20.4 | 258 | ± | 23.6 | 264 | ± | 20.3 | 258 | ± | 24.3 | 255 | ± | 23.1 | 255 | ± | 25.7 | 251 | ± | 22.7 | |
| **20:3n3** | <LLOQ |  |  | <LLOQ |  |  | <LLOQ |  |  | <LLOQ |  |  | <LLOQ |  |  | <LLOQ |  |  | <LLOQ |  |  | <LLOQ |  |  | |
| **20:4n3** | 3.91 | ± | 0.59 | 3.84 | ± | 0.53 | 3.88 | ± | 0.35 | 4.29 | ± | 0.39 | 3.53 | ± | 0.34 | 3.62 | ± | 0.31 | 3.20 | ± | 0.29 | 3.57 | ± | 0.37 | |
| **20:5n3** | 22.3 | ± | 2.76 | 26.3 | ± | 2.61 | 49.8 | ± | 9.99 | 105.9 | ± | 13.3 | 66.4 | ± | 7.22 | 64.6 | ± | 4.52 | 52.4 | ± | 4.76 | 40.9 | ± | 3.78 | |
| **22:0** | 23.1 | ± | 1.64 | 20.9 | ± | 1.32 | 22.0 | ± | 1.80 | 22.4 | ± | 1.24 | 21.6 | ± | 1.87 | 23.3 | ± | 1.89 | 23.1 | ± | 1.89 | 22.5 | ± | 1.85 | |
| **22:1n9** | 6.71 | ± | 1.20 | 4.66 | ± | 0.82 | 5.33 | ± | 1.32 | 3.50 | ± | 0.42 | 5.78 | ± | 1.40 | 4.66 | ± | 0.63 | 5.59 | ± | 0.86 | 3.31 | ± | 0.54 | |
| **22:2n6** | <LLOQ |  |  | <LLOQ |  |  | <LLOQ |  |  | <LLOQ |  |  | <LLOQ |  |  | <LLOQ |  |  | <LLOQ |  |  | <LLOQ |  |  | |
| **22:4n6** | 11.2 | ± | 0.92 | 10.6 | ± | 0.76 | 10.9 | ± | 0.78 | 10.8 | ± | 0.57 | 10.0 | ± | 0.81 | 10.5 | ± | 0.68 | 10.8 | ± | 1.03 | 10.1 | ± | 0.76 | |
| **22:5n6** | 6.06 | ± | 0.74 | 5.75 | ± | 0.65 | 6.23 | ± | 0.79 | 5.90 | ± | 0.45 | 5.47 | ± | 0.67 | 5.92 | ± | 0.61 | 5.88 | ± | 0.75 | 5.59 | ± | 0.54 | |
| **22:5n3** | 23.7 | ± | 1.90 | 23.0 | ± | 1.35 | 24.2 | ± | 1.88 | 25.3 | ± | 1.41 | 23.1 | ± | 1.99 | 28.2 | ± | 1.86 | 28.5 | ± | 2.53 | 29.6 | ± | 2.19 | |
| **24:0** | 18.6 | ± | 1.30 | 17.3 | ± | 1.35 | 17.8 | ± | 1.52 | 17.0 | ± | 1.42 | 17.1 | ± | 1.59 | 18.9 | ± | 1.73 | 19.1 | ± | 1.86 | 19.0 | ± | 1.59 | |
| **22:6n3** | 49.9 | ± | 5.74 | 47.2 | ± | 4.48 | 47.9 | ± | 5.14 | 47.4 | ± | 4.43 | 45.8 | ± | 5.11 | 48.9 | ± | 4.93 | 47.3 | ± | 4.92 | 48.5 | ± | 4.48 | |
| **24:1n9** | 38.3 | ± | 4.32 | 34.0 | ± | 1.89 | 34.3 | ± | 2.52 | 35.4 | ± | 2.92 | 34.3 | ± | 2.63 | 38.6 | ± | 3.02 | 38.7 | ± | 3.42 | 39.8 | ± | 3.20 | |

| **Table S3:** Full plasma fatty acid concentrations [µg/mL] after single-dose ingestion of the DHA concentrate. All results are expressed as mean ± SE (n = 12). | | | | | | | | | | | | | | | | | | | | | | | | |
| --- | --- | --- | --- | --- | --- | --- | --- | --- | --- | --- | --- | --- | --- | --- | --- | --- | --- | --- | --- | --- | --- | --- | --- | --- |
|  | | | |  |  |  |  |  |  |  |  |  |  |  |  |  |  |  |  |  |  |  |  |  |
|  | **t0** |  |  | **t2** |  |  | **t4** |  |  | **t6** |  |  | **t8** |  |  | **t24** |  |  | **t48** |  |  | **t72** |  |  |
| **fatty acid** | **mean** | **±** | **SE** | **mean** | **±** | **SE** | **mean** | **±** | **SE** | **mean** | **±** | **SE** | **mean** | **±** | **SE** | **mean** | **±** | **SE** | **mean** | **±** | **SE** | **mean** | **±** | **SE** |
| **10:0** | <LLOQ |  |  | <LLOQ |  |  | <LLOQ |  |  | <LLOQ |  |  | <LLOQ |  |  | <LLOQ |  |  | <LLOQ |  |  | <LLOQ |  |  |
| **11:0** | <LLOQ |  |  | <LLOQ |  |  | <LLOQ |  |  | <LLOQ |  |  | <LLOQ |  |  | <LLOQ |  |  | <LLOQ |  |  | <LLOQ |  |  |
| **12:0** | 3.51 | ± | 0.91 | 9.31 | ± | 2.07 | 11.01 | ± | 1.98 | 11.98 | ± | 2.90 | 6.50 | ± | 1.73 | 2.74 | ± | 0.41 | 3.72 | ± | 0.61 | 3.03 | ± | 0.43 |
| **13:0** | <LLOQ |  |  | <LLOQ |  |  | <LLOQ |  |  | <LLOQ |  |  | <LLOQ |  |  | <LLOQ |  |  | <LLOQ |  |  | <LLOQ |  |  |
| **14:0** | 37.1 | ± | 7.49 | 68.7 | ± | 12.2 | 80.8 | ± | 12.6 | 91.5 | ± | 18.0 | 62.7 | ± | 13.2 | 35.5 | ± | 5.27 | 38.7 | ± | 6.64 | 40.7 | ± | 5.67 |
| **14:1n5** | 2.87 | ± | 0.87 | 5.17 | ± | 1.23 | 6.72 | ± | 1.30 | 7.52 | ± | 1.75 | 5.48 | ± | 1.51 | 2.45 | ± | 0.50 | 2.46 | ± | 0.56 | 2.82 | ± | 0.48 |
| **15:0** | 8.45 | ± | 0.93 | 11.8 | ± | 1.30 | 12.9 | ± | 1.34 | 14.5 | ± | 2.06 | 11.1 | ± | 1.42 | 9.18 | ± | 0.87 | 9.15 | ± | 0.91 | 10.5 | ± | 1.04 |
| **15:1n5** | <LLOQ |  |  | <LLOQ |  |  | <LLOQ |  |  | <LLOQ |  |  | <LLOQ |  |  | <LLOQ |  |  | <LLOQ |  |  | <LLOQ |  |  |
| **16:0** | 698 | ± | 75.1 | 797 | ± | 86.0 | 845 | ± | 87.1 | 886 | ± | 112 | 776 | ± | 93.9 | 668 | ± | 49.6 | 636 | ± | 50.8 | 677 | ± | 60.2 |
| **16:1n7** | 72.5 | ± | 11.6 | 75.2 | ± | 11.3 | 78.3 | ± | 11.8 | 81.1 | ± | 15.4 | 68.0 | ± | 13.1 | 62.4 | ± | 8.01 | 56.8 | ± | 6.30 | 61.5 | ± | 6.97 |
| **17:0** | 8.31 | ± | 0.81 | 10.1 | ± | 0.98 | 10.9 | ± | 1.02 | 11.6 | ± | 1.39 | 9.92 | ± | 1.11 | 8.64 | ± | 0.65 | 8.25 | ± | 0.70 | 9.21 | ± | 0.73 |
| **17:1n7** | <LLOQ |  |  | <LLOQ |  |  | <LLOQ |  |  | <LLOQ |  |  | <LLOQ |  |  | <LLOQ |  |  | <LLOQ |  |  | <LLOQ |  |  |
| **18:0** | 206 | ± | 15.3 | 242 | ± | 18.0 | 252 | ± | 17.7 | 270 | ± | 25.8 | 241 | ± | 20.1 | 208 | ± | 10.6 | 199 | ± | 13.9 | 209 | ± | 16.3 |
| **18:1n9** | 698 | ± | 70.7 | 738 | ± | 76.5 | 757 | ± | 71.9 | 755 | ± | 84.7 | 634 | ± | 68.0 | 604 | ± | 43.2 | 550 | ± | 39.2 | 583 | ± | 53.6 |
| **18:1n7** | 60.9 | ± | 6.44 | 67.2 | ± | 6.44 | 69.1 | ± | 6.16 | 71.2 | ± | 7.21 | 60.0 | ± | 4.93 | 59.9 | ± | 3.78 | 55.8 | ± | 3.96 | 60.1 | ± | 5.42 |
| **18:2n6** | 725 | ± | 47.0 | 726 | ± | 44.1 | 733 | ± | 34.8 | 743 | ± | 50.6 | 718 | ± | 35.6 | 685 | ± | 28.0 | 709 | ± | 42.0 | 707 | ± | 46.7 |
| **18:3n6** | 11.6 | ± | 1.95 | 11.5 | ± | 1.99 | 11.5 | ± | 1.99 | 11.4 | ± | 2.18 | 11.3 | ± | 2.30 | 9.78 | ± | 1.35 | 9.39 | ± | 1.42 | 8.81 | ± | 1.26 |
| **19:0** | 0.68 | ± | 0.05 | 0.82 | ± | 0.06 | 0.93 | ± | 0.08 | 0.96 | ± | 0.07 | 0.84 | ± | 0.05 | 0.71 | ± | 0.05 | 0.73 | ± | 0.06 | 0.83 | ± | 0.05 |
| **18:3n3** | 14.6 | ± | 1.41 | 15.5 | ± | 1.46 | 15.7 | ± | 1.25 | 15.8 | ± | 1.53 | 12.8 | ± | 1.12 | 13.7 | ± | 0.97 | 12.8 | ± | 1.16 | 15.7 | ± | 1.43 |
| **18:4n3** | <LLOQ |  |  | <LLOQ |  |  | <LLOQ |  |  | <LLOQ |  |  | <LLOQ |  |  | <LLOQ |  |  | <LLOQ |  |  | <LLOQ |  |  |
| **20:0** | 6.51 | ± | 0.41 | 7.08 | ± | 0.42 | 7.00 | ± | 0.34 | 7.16 | ± | 0.52 | 6.90 | ± | 0.37 | 6.60 | ± | 0.26 | 6.58 | ± | 0.42 | 6.75 | ± | 0.51 |
| **20:1n9** | 6.18 | ± | 0.54 | 6.14 | ± | 0.51 | 6.05 | ± | 0.42 | 5.94 | ± | 0.55 | 5.19 | ± | 0.41 | 5.28 | ± | 0.33 | 4.69 | ± | 0.30 | 5.05 | ± | 0.49 |
| **20:2n6** | 6.07 | ± | 0.52 | 6.06 | ± | 0.45 | 6.04 | ± | 0.36 | 6.14 | ± | 0.54 | 5.58 | ± | 0.44 | 5.79 | ± | 0.35 | 5.46 | ± | 0.41 | 5.71 | ± | 0.48 |
| **20:3n9** | 4.48 | ± | 0.72 | 4.55 | ± | 0.73 | 4.54 | ± | 0.67 | 4.53 | ± | 0.73 | 4.45 | ± | 0.70 | 4.31 | ± | 0.64 | 3.89 | ± | 0.58 | 3.99 | ± | 0.65 |
| **20:3n6** | 51.1 | ± | 4.53 | 51.0 | ± | 3.74 | 51.7 | ± | 3.58 | 53.0 | ± | 4.85 | 53.4 | ± | 4.56 | 53.9 | ± | 3.92 | 52.4 | ± | 4.03 | 54.4 | ± | 4.33 |
| **21:0** | <LLOQ |  |  | <LLOQ |  |  | <LLOQ |  |  | <LLOQ |  |  | <LLOQ |  |  | <LLOQ |  |  | <LLOQ |  |  | <LLOQ |  |  |
| **20:4n6** | 197 | ± | 20.8 | 199 | ± | 21.4 | 201 | ± | 18.3 | 205 | ± | 18.8 | 207 | ± | 15.8 | 203 | ± | 15.4 | 200 | ± | 21.3 | 204 | ± | 21.6 |
| **20:3n3** | <LLOQ |  |  | <LLOQ |  |  | <LLOQ |  |  | <LLOQ |  |  | <LLOQ |  |  | <LLOQ |  |  | <LLOQ |  |  | <LLOQ |  |  |
| **20:4n3** | 2.20 | ± | 0.41 | 2.26 | ± | 0.34 | 2.26 | ± | 0.35 | 2.23 | ± | 0.41 | 2.16 | ± | 0.38 | 2.26 | ± | 0.34 | 2.00 | ± | 0.28 | 2.37 | ± | 0.33 |
| **20:5n3** | 17.5 | ± | 1.98 | 16.8 | ± | 2.01 | 16.7 | ± | 1.80 | 17.3 | ± | 2.08 | 17.5 | ± | 2.10 | 17.7 | ± | 1.70 | 16.6 | ± | 1.61 | 16.8 | ± | 1.83 |
| **22:0** | 17.8 | ± | 1.11 | 18.0 | ± | 1.03 | 18.1 | ± | 0.97 | 17.8 | ± | 1.00 | 17.7 | ± | 0.79 | 18.4 | ± | 0.94 | 18.1 | ± | 1.16 | 18.3 | ± | 1.20 |
| **22:1n9** | 2.47 | ± | 0.41 | 2.89 | ± | 0.54 | 1.99 | ± | 0.25 | 2.29 | ± | 0.41 | 2.33 | ± | 0.27 | 1.96 | ± | 0.30 | 2.12 | ± | 0.33 | 2.89 | ± | 0.49 |
| **22:2n6** | <LLOQ |  |  | <LLOQ |  |  | <LLOQ |  |  | <LLOQ |  |  | <LLOQ |  |  | <LLOQ |  |  | <LLOQ |  |  | <LLOQ |  |  |
| **22:4n6** | 7.44 | ± | 0.66 | 7.79 | ± | 0.66 | 7.96 | ± | 0.57 | 8.03 | ± | 0.69 | 7.81 | ± | 0.63 | 7.55 | ± | 0.55 | 7.25 | ± | 0.77 | 7.55 | ± | 0.68 |
| **22:5n6** | 4.06 | ± | 0.55 | 4.08 | ± | 0.53 | 4.28 | ± | 0.49 | 4.36 | ± | 0.57 | 4.16 | ± | 0.49 | 4.40 | ± | 0.50 | 4.16 | ± | 0.53 | 4.35 | ± | 0.51 |
| **22:5n3** | 17.4 | ± | 1.63 | 17.8 | ± | 1.60 | 17.5 | ± | 1.47 | 18.4 | ± | 1.69 | 17.4 | ± | 1.49 | 17.3 | ± | 1.48 | 16.0 | ± | 1.38 | 16.4 | ± | 1.48 |
| **24:0** | 16.6 | ± | 1.09 | 16.6 | ± | 1.05 | 16.4 | ± | 0.96 | 16.3 | ± | 0.98 | 16.8 | ± | 0.86 | 17.2 | ± | 1.05 | 17.1 | ± | 1.16 | 17.4 | ± | 1.14 |
| **22:6n3** | 37.9 | ± | 4.25 | 41.4 | ± | 3.84 | 46.2 | ± | 5.66 | 80.0 | ± | 12.6 | 58.0 | ± | 6.84 | 51.3 | ± | 3.67 | 49.7 | ± | 4.39 | 51.7 | ± | 5.38 |
| **24:1n9** | 31.4 | ± | 2.18 | 30.0 | ± | 2.07 | 30.0 | ± | 1.68 | 29.0 | ± | 1.63 | 30.0 | ± | 1.34 | 31.2 | ± | 1.50 | 31.7 | ± | 1.99 | 32.7 | ± | 2.43 |
